# Reversible pH-Sensitive Chitosan-Based Hydrogels. Influence of Dispersion Composition on Rheological Properties and Sustained Drug Delivery

**DOI:** 10.3390/polym10040392

**Published:** 2018-04-01

**Authors:** Nieves Iglesias, Elsa Galbis, Concepción Valencia, M.-Violante de-Paz, Juan A. Galbis

**Affiliations:** 1 Departamento de Química Orgánica y Farmacéutica, Facultad de Farmacia, Universidad de Sevilla, 41012 Sevilla, Spain; nievesiglesias@us.es (N.I.); elsa@us.es (E.G.); jgalbis@us.es (J.A.G.); 2 Departamento de Ingeniería Química, Campus de “El Carmen”, Universidad de Huelva, 21071 Huelva, Spain; barragan@diq.uhu.es; 3 Pro2TecS—Chemical Process and Product Technology Research Center, Universidad de Huelva, 21071 Huelva, Spain

**Keywords:** ionic cross-linking, eco-friendly formulations, thermal transition sol-gel, drug delivery systems, MTDSC, DSC

## Abstract

The present work deals with the synthesis of micro-structured biomaterials based on chitosan (CTS) for their applications as biocompatible carriers of drugs and bioactive compounds. Twelve dispersions were prepared by means of functional cross-linking with tricarballylic acid (TCA); they were characterized by Fourier transform infrared spectroscopy (FT-IR), modulated temperature differential scanning calorimetry (MTDSC) and scanning electron microscopy (SEM), and their rheological properties were studied. To the best of the authors’ knowledge, no study has been carried out on the influence of CTS concentration, degree of cross-linking and drug loading on chitosan hydrogels for drug delivery systems (DDS) and is investigated herein for the first time. The influence of dispersion composition (polymer concentration and degree of cross-linking) revealed to exert a marked impact on its rheological properties, going from liquid-like to viscoelastic gels. The release profiles of a model drug, diclofenac sodium (DCNa), as well as their relationships with polymer concentration, drug loading and degree of cross-linking were evaluated. Similar to the findings on rheological properties, a wide range of release profiles was encountered. These formulations were found to display a well-controlled drug release strongly dependent on the formulation composition. Cumulative drug release under physiological conditions for 96 h ranged from 8% to 67%. For comparative purpose, Voltaren emulgel^®^ from Novartis Pharmaceuticals was also investigated and the latter was the formulation with the highest cumulative drug release (85%). Some formulations showed similar spreadability values to the commercial hydrogel. The comparative study of three batches confirmed the reproducibility of the method, leading to systems particularly suitable for their use as drug carriers.

## 1. Introduction

Hydrogels are currently under investigation as matrices for the controlled release of bioactive molecules, in particular drugs and proteins, and for the encapsulation of living cells. In general terms, hydrogels are constituted by cross-linked polymer networks that have a high amount of hydrophilic domains with affinity for water. Among the favorable features of these systems, the similarity between their physical properties and those of living tissues, such as low interfacial tension with water or biological fluids, can be highlighted [1,2]. Likewise, the elastic nature of hydrated hydrogels minimizes irritation of surrounding tissues after implantation. Moreover, the low interfacial tension between the hydrogel surface and body fluid lessens protein adsorption and cell adhesion, which reduces the chances of a negative immune reaction [3]. For biomedical applications, gels are often required to degrade under physiological conditions and lead to the disintegration of the three-dimensional structure, preferably in harmless products, to ensure good biocompatibility of the hydrogel [2].

Being a non-toxic, biocompatible and biodegradable polymer, chitosan (CTS) and its hydrogels have been widely used as biomaterials for drug delivery, gene delivery, and tissue engineering and, in recent years, numerous scientific papers have been published on a wide variety of biomedical applications [4,5]. The polysaccharide CTS, a weak cationic polysaccharide composed of randomly distributed β-(1–4)-linked d-glucosamine and *N*-acetyl-d-glucosamine repeating units, is a copolymer prepared from renewable resources. It can be obtained from the partial deacetylation of the second most important natural polymer in the world: chitin or poly(*N*-acetyl-β-d-glucosamine). Its abundance in marine crustacean such as shrimp and crabs makes it a commercial product with global impact in polymer science. 

CTS hydrogels can be prepared via physical association (ionic crosslinking) [5,6,7,8,9], coordination with metal ions [10], or irreversible/chemical cross-linking between chitosan and the crosslinker [11,12,13]. The chemical and/or physical linkages will prevent the networks from dissolving. Interestingly, and regarding the release of bioactive molecules, the reversible nature of ionically cross-linked networks is useful for their potential applications as drug delivery systems. Therefore, once the release of the drug in the medium have been accomplished, the formulations can subsequently disintegrate into biocompatible components that will then be metabolized and eliminated from the body [2].

Few anions such as citrate salts, dextran sulfate, glycerol-mono or diphosphates, polyphosphates, glucose-phosphates and other polyol-phosphates have been tested in the formation of ionically crosslinked chitosan hydrogels [2,6,7,8,14]. However, a lack of study on carboxylate derivatives as cross-linkers is found apart from few polyanionic materials such as poly(methacrylic acid) and alginates, in which an additional secondary force, the chain entanglement is involved [7,10,15,16,17].

We describe herein a highly efficient strategy for the preparation of a new batch of pH-sensitive reversible chitosan hydrogels, which are endowed with controlled release properties under physiological conditions. The prepared chitosan hydrogels are stabilized by ionic crosslinking with propane-1,2,3-tricarboxylic acid (tricarballylic acid, TCA). To the best of the authors’ knowledge, no study has been carried out on the influence of the CTS concentration, degree of cross-linking and drug loading on the rheological properties and drug release profiles of CTS-based hydrogels and this is explored herein for the first time. The influence of the concentration of CTS (from 2% to 4%) and the degree of cross-linking (from 0% to 15%) on the rheological properties of the systems is investigated. The anionic model drug, diclofenac sodium, is loaded in CTS formulations in which the concentration of CTS (from 2% to 4%), the degree of cross-linking (from 0% to 15%) and the drug loading (from 0.5% to 2%) are set to investigate the effect of those parameters on the drug release. Thus, a wide range of systems is studied and marked differences encountered, not only in their rheological properties but also in their release profiles.

## 2. Materials and Methods

All the chemicals used were purchased from Sigma-Aldrich (Madrid, Spain) and used as received. A commercial chitosan (CTS) from Sigma-Aldrich with a deacetylation degree of 75% was chosen. The molecular weight of the CTS used in the present work was determined by viscometric analysis. Its viscosity was measured in a buffered solution of 0.5 M acetic acid—0.5 M sodium acetate solution at 25.0 ± 0.1 °C using an Anton Paar AMVn automated microviscometer (Ashland, VA, US). The viscometric constants *a* and *K* in the Mark-Houwink equation were previously determined for this solvent—CTS system and found to be *a* = 0.59 and *K* = 0.119 cm^3^·g^−1^ [18]. The weight of the CTS used in the present work was calculated means of the Mark-Houwink equation ([η] = 3.385 dL/g) and its value was 299 kDa. The acetate buffer at pH 5.5 (25 °C) for release assays was prepared in-house, with pH variations vs. temperature of ±0.1 from 5 to 50 °C. Voltaren emulgel^®^ (Novartis Farmaceutica, Barcelona, Spain) was purchased from a licensed drugstore (Seville, Spain). 

To investigate the chemical interaction between chitosan and tricarballylic acid (TCA) in aqueous media, IR spectra of chitosan, tricarballylic acid and CTS-TCA conjugate were recorded on a Jasco FT/IR 4200 spectrometer (Great Dunmow, Essex, UK) equipped with attenuated total single reflection (ATR) accessory in the range between 4000 and 600 cm^−1^.

Measurement of UV and visible light absorbance was performed with an Agilent 8453 UV-Visible spectrophotometer (Palo Alto, CA, USA), equipped with diode array detection (DAD); the data were the result of at least three measurements.

The phase transitions exhibited by the new prepared cross-linked hydrogels were examined by modulated temperature differential scanning calorimetry (MTDSC), using a TA DSC Q-200 Instrument (calibrated with indium, Cerdanyola del Valles, Spain) and a refrigerated cooling system (RCS) to ensure proper temperature cycling. Accurately weighed 3–6 mg samples were then hermetically sealed into aluminum DSC pans, and the equipment was operated in modulation mode. Calorimetric scans were carried out at a scanning rate of 2 °C/min under nitrogen atmosphere over an appropriate temperature range (from 0 to 140 °C). Modulation amplitude was ±0.159 °C every 30 s. 

The morphologies of selected samples were examined by Scanning Electron Microscopy (SEM). Before SEM observations, the hydrogel scaffold was directly frozen at −20 °C for 3 h, then at −80 °C for 24 h. The samples were then lyophilized by freeze drying for 24 h. Finally, the dry hydrogel was fixed on aluminum stubs, coated with a thickness of about 25 nm of gold, and analyzed at the Electron Microscopy Division of the Scientific Integrated Services (SC-ICYT) of the University of Cádiz (Spain) using a field emission scanning electron microscope FEI Nova NanoSEM 450 (Hillsboro, Oregon, US) operated at 5 kV.

To determine of spreadability of a specific formulation, the selected hydrogel (300 mg) was placed in the center of a glass plate (20 cm × 20 cm) and the sample was covered by another plate of similar dimensions. A weight of 30 g was carefully placed in the center of the upper cover avoiding the sliding between the plates. The spread area (diameter, in cm) of the gel was measured after 1 min and 30 min. 

### 2.1. Preparation of Hydrogels from Crosslinked Chitosan-Tricarballylic Acid (CTS_x_-TCA_y_)

Twelve systems named **CTS_x_**-**TCA_y_** were prepared with CTS of molecular weight ranging from 190 to 375 kDa, (based on viscosity values). The targeted final CTS concentrations were 2%, 3% or 4% *w*/*w* and the degree of crosslinking 0%, 5%, 10% or 15%. The later parameter was fixed by the amount of tricarballylic acid (TCA) added to the formulation. In Table 1 and along the text “x” denotes CTS concentration (% *w*/*w*) and “y” denotes the degree of cross-linking in the hydrogel.

A typical procedure for the preparation of aqueous cross-linked chitosan-carballylic acid conjugates at 3% *w*/*w* polymer concentration and 5% of degree of crosslinking (**CTS_3_-TCA_5_**) is summarized next: chitosan with a deacetylation degree of 75% (chitosan, 0.3 g, 1.31 mmol of free amine groups) was charged in a round-bottom flask provided with a stirrer bar; then, an aqueous solution of tricarballylic acid (TCA, 0.38 mL, 10mg/mL, 0.02 mmol), a solution of acetic acid (HAc, 0.1 mL, 52% *w*/*v*) and double-distilled water (up to a final weight of 10 g, and final polymer concentration of 3% *w*/*w*) were added in sequence. The mixture was stirred to homogenization at 40 °C during 1.5 h. The solution was cooled and the stirring proceeded overnight at 25 °C. Three different batches of these conjugates were synthesized for comparative purposes. 

**CTS_x_-TCA_y_** hydrogels were rheologically characterized in a controlled-strain (ARES, Rheometric Scientific, Surrey, UK) rheometer, using a serrated plate-plate (25 mm diameter, 1 mm gap) geometry. Small amplitude oscillatory shear (SAOS) tests were carried out inside the linear viscoelastic region in a frequency range of 0.03–100 rad/s at 25 °C. Stress sweep tests were previously performed to determine the linear viscoelastic regime. Viscous flow tests were also made by applying a stepped shear rate ramp in a shear rate range of 0.06–100 s^−1^ at 25 °C. Each fresh sample was tested at least in duplicate.

### 2.2. Preparation of Diclofenac Sodium Loaded Formulations from Crosslinked Chitosan-Conjugates

Eight systems named **CTS_x_-TCA_y_-DCNa_z_** were prepared with targeted final CTS concentrations 2%, 3% or 4% *w*/*w* and degree of crosslinking 0%, 5%, 10% or 15%. Diclofenac sodium was loaded into the hydrogel formulation so that the final *w*/*w* concentrations ranged from 0.5% to 2%. In Table 2 and along the text “x” denotes CTS concentration (% *w*/*w*), “y” denotes the degree of cross-linking and “z” denotes DCNa concentration (% *w*/*w*).

### 2.3. Diclofenac Sodium Release Studies

**Figure 1:**
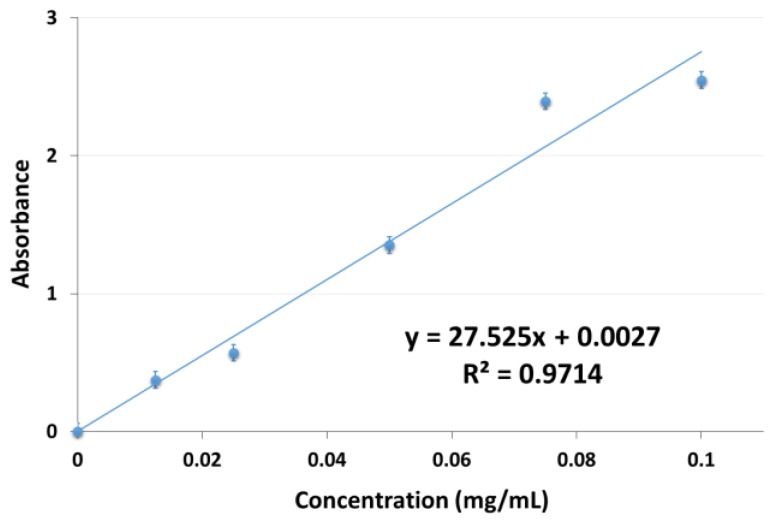
Calibration curve of diclofenac sodium at 280 nm (UV-Vis spectroscopy) at 25 °C.

Prior to the release analyses, a calibration curve of diclofenac sodium was made with DCNa standard solutions (in buffered acetate solutions at pH 5.5) at 280 nm (Figure 1). For the calibration, a stock solution of sodium diclofenac at 100 µg/mL concentration was used. By dilution, 4 solutions were prepared with the following concentrations: 75, 50, 25, and 12.5 µg/mL.

The selected hydrogel (0.1 g) was transferred to a dialysis bag (molecular weight cut-off: 8000–14,000 Da) and then immersed in 20 mL of acetate buffer solution (pH = 5.5). Experiments were performed at 37 °C in a shaker incubator (Heidolph Unimax 1010-Heidolph Inkubator 1000, Schwabach, Germany). At pre-designed time intervals, aliquots of 1 mL were taken from the release medium and the amount of DCNa released was determined by UV-Vis spectroscopy at 280 nm. 1 mL of pre-heated buffer solution was added to the release medium to maintain a constant volume. DCNa release experiments were performed in triplicates. Analogous experiments were performed under the same conditions with Voltaren emulgel^®^ (0.1 g).

## 3. Results and Discussion

### 3.1. Cross-Linked Chitosan-Tricarballylic Acid (CTSx TCAy) Hydrogels

To form stabilizing linkages, CTS-based hydrogels have amino and hydroxyl functional groups that allow linking between the chain and the cross-linker to prevent gel dissolution. Hydrogel binding could be accomplished by reversible cross-linking reactions, for example by means of ionic cross-linkers negatively ionized at physiological pH such as citrate salts [6], glycerol-mono or diphosphates [14], polyphosphates [8], glucose-phosphates, and other polyol-phosphates [7]. This method can sufficiently restrain hydrogel structure, but the physical associations are reversible bonds, whereas the covalent cross-linkages between polymer chains are not. This distinction is relevant for the drug release kinetics and further biodegradation of drug-delivery-system (DDS) hydrogels [3].

In the present work, the preparation of a batch of new DDS from CTS-based reversible cross-linked hydrogels has been carried out. Propane-1,2,3-tricarboxylic acid (tricarballylic acid, TCA) has been the cross-linker of choice due to its hydrophilic character, biocompatibility, and symmetry that can facilitate the formation of homogeneous aqueous dispersions with reproducible physical properties. In CTS/TCA systems, several types of interactions may be involved during the gelation process: (1) electrostatic attraction between CTS ammonia groups and TCA carboxylate groups: since p*K*a of CTS and TCA are 6.5 and 4.1, respectively, the amine and carboxylic acid groups will be mainly ionized at the pH of the formulations (pH ≈ 5.2–5.3), i.e., when the pH is more than one unit under or one unit over their p*K*a, respectively. Thus, electrostatic attractions between the ammonia groups from chitosan and the carboxylate groups from the tricarballylic acid are expected to be significant leading to the stability of the hydrogel systems at those pHs; (2) Other non-covalent physical associations or secondary forces such as hydrogen bonding as a consequence of reduced electrostatic repulsion after neutralization of CTS with TCA and physical entanglements [19]. 

pH Values from 5.5 to 7.0 are within the so-called physiological values in human beings; for example, slightly acidic microenvironments are found in mucous membranes and other topical areas. However, maintaining acid-base balance is critical for the survival of living species since cellular processes are highly sensitive to changes in proton concentrations. Although in humans, pH varies within a narrow range (in the blood between pH 7.35 and 7.45), local deviations from the systemic pH are often caused by pathologies, such as cancer, inflammation, infection, ischemia, renal failure or pulmonary disease [20]. Consequently, drug delivery systems active at acidic pH, such as the formulations studied herein, could find significant applications in a wide field of pathologies and locations.

Twelve systems named **CTS_x_-TCA_y_** were prepared with CTS of molecular weight ranging from 190 to 375 kDa, (based on viscosity values). The targeted final CTS concentrations, “x”, were 2%, 3% or 4% *w*/*w* and the degree of cross-linking, “y” 0%, 5%, 10% or 15% (Table 1). The degree of cross-linking was calculated based on the number of mequiv of free amine groups present in the CTS used and the number of mequiv of carboxylic acid groups present in the amount of the cross-linker added. All the samples displayed a clear and slightly-colored appearance with the absence of solid particles in suspension and the pH of the dispersions ranged from 5.2 to 5.3. An in-depth analysis on their rheological properties was conducted to determine the influence of the hydrogel composition on their viscoelastic properties and viscosities. Likewise, some additional studies were carried out by Fourier transform infrared spectroscopy (FT-IR), scanning electron microscopy (SEM), and differential scanning calorimetry (DSC) and the results are discussed below.

#### 3.1.1. Fourier Transform Infrared Spectroscopy (FT-IR)

**Figure 2:**
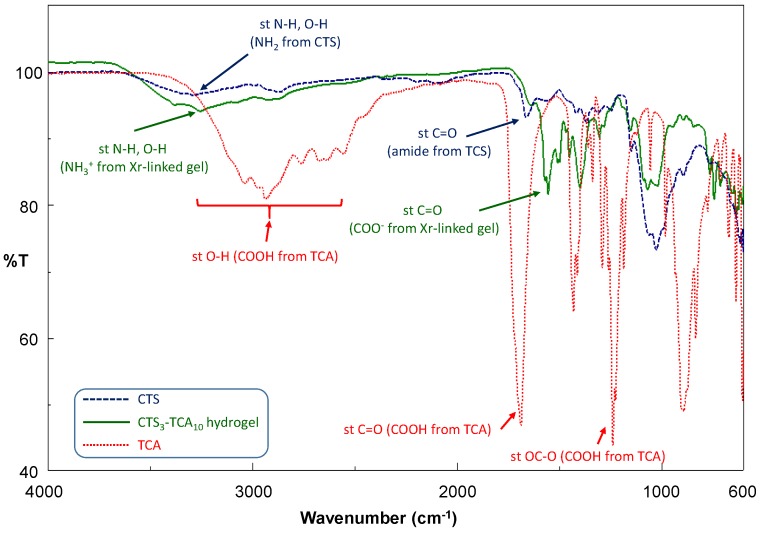
FT-IR spectra of CTS (blue); freeze-dried CTS_3_-TCA_10_ hydrogel (green) and TCA (red).

To examine the chemical interactions between CTS and the cross-linker in the hydrogels, IR spectra of CTS, TCA, and freeze-dried CTS-TCA conjugates were recorded. Figure 2 shows the FTIR spectra of CTS, TCA and the freeze-dried hydrogel **CTS_3_-TCA_10_** and they were in concurrence with their chemical structure. Thus, from the CTS spectra, the O-H bonds from hydroxyl groups provide a broad band centered at 3288 cm^−1^, which is overlapped with the stretching bands corresponding to the N–H bonds from amine and amide groups. The absorption band centered at 1064 cm^−1^ can also be observed (st C–OH). The acetamido groups in the *N*-acetyl-d-glucosamine repeating units are responsible of the characteristic band at 1664 cm^−1^, attributed to the stretching vibration of the C=O amide (amide I band). From the TCA FTIR spectrum two significant bands correlated to the carboxylic acid groups of the cross-linker can be found: the first one, a broad band centered at 2937 cm^−1^ displays the characteristic feature of those associated to the stretching of O–H bonds from carboxylic acids, and the second one, an intense stretching band due to the C=O group, appeared at 1687 cm^−1^. The most intense band in the spectrum is due to the stretching vibration of OC–O bond from the carboxylic acid groups at 1238 cm^−1^. None of them are present in sample **CTS_3_-TCA_10_** in which the acid-base reaction between CTS and TCA had taken placed. Consequently, a new band at 1555 cm^−1^ (stretching band of C=O in carboxylate ions), has emerged as well as a shift and gain in intensity of st N–H band, in this case correlated with ammonia ions. These findings were consistent with the effective preparation of ionic cross-linked CTS-based hydrogels.

#### 3.1.2. Rheological Characterization of CTS_x_-TCA_y_ Hydrogels

**Figure 3:**
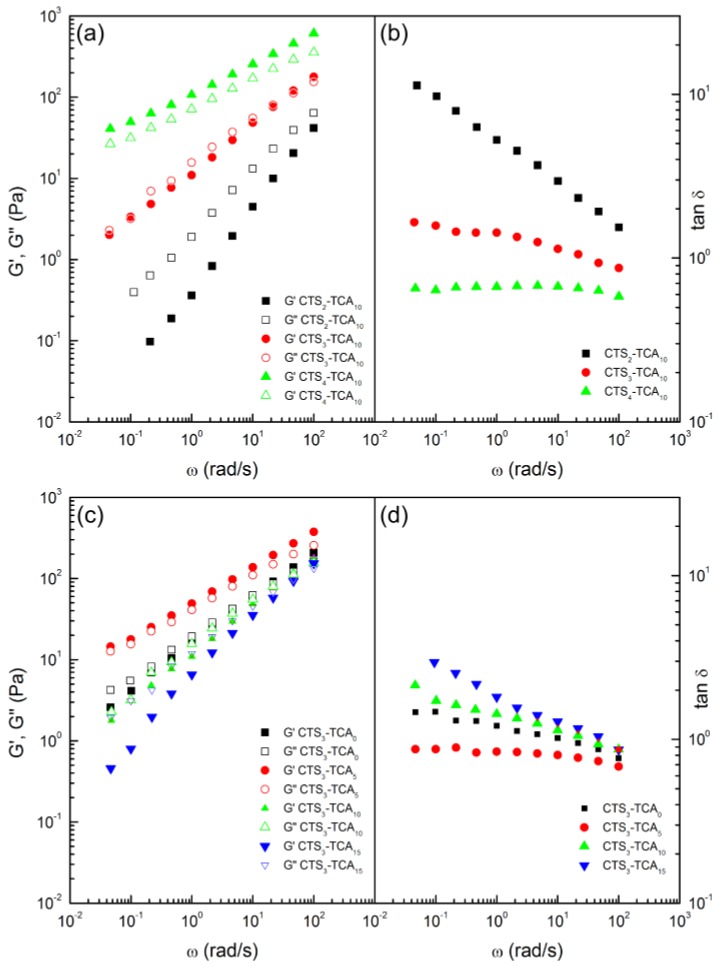
Frequency dependence of the storage, *G*’, and loss, *G*”, moduli and the loss tangent for **CTS_x_-TCA_y_** hydrogels as function of CTS (**a**,**b**) and degree of cross-linking (**c**,**d**).

Figure 3a–d illustrate the evolution of SAOS functions with frequency, at 25 °C, inside the linear viscoelastic range for **CTS_x_-TCA_y_** hydrogels as function of CTS concentration and degree of cross-linking based on TCA, respectively. As can be observed, different responses were obtained depending on CTS concentration and degree of cross-linker. Regarding the influence of CTS concentration, at low CTS concentration (**CTS_2_-TCA_10_**) the values of the loss modulus (*G*″) are significantly higher than those found for the storage modulus (*G*′), and the formulations are not really resulting in gels but liquid-like viscoelastic dispersions. A tendency to reach a crossover point between these moduli was observed at high frequencies. This corresponds to the dynamic characteristics of a viscoelastic polymer fluid without entanglements. At an intermediate CTS concentration (**CTS_3_-TCA_10_**), *G*′ coincides with *G*″ over a wide frequency range. This power law behavior suggests a gel-like behavior quite close to a critical gel [21]. On the other hand, at high CTS concentration (**CTS_4_-TCA_10_**), *G*′ becomes higher than *G*″ and parallel in the whole frequency range. This indicates the formation of viscoelastic gels. 

As well known [20], the gel strength of biopolymer dispersed systems, from dilute solutions to crosslinked gels, can be quantified from SAOS measurements as a function of the *G*’ and *G*″ frequency dependence, i.e., the slopes of *G*’ and *G*” versus frequency plots, and the relative values of both viscoelastic functions, i.e., the relative elasticity, expressed in terms of the loss tangent (tan δ = *G*″/*G*’). Regarding the evolution of the loss tangent with frequency (Figure 3b), the **CTS_4_-TCA_10_** hydrogel shows the lowest values of the loss tangent indicating a higher relative elasticity due to the high level of cross-linking and physical entanglements, followed by **CTS_3_-TCA_10_** hydrogel. On the other hand, **CTS_2_-TCA_10_** displays the highest values of the loss tangent, higher than 1 in the whole frequency range indicating a essentially viscous behavior characteristic of polymer fluids without significant formation of physical entanglements [22]. The influence of cross-linker concentration on the mechanical spectra, obtained from SAOS measurements and loss tangent of **CTS_x_-TCA_y_** hydrogels are presented in Figure 3c,d, respectively. At high TCA concentration (**CTS_3_-TCA_15_**), the values of *G*” are higher than those found for *G*’ and a tendency to reach a crossover point between G’ and *G*” was found at high frequencies, which mainly exhibits a viscous response. This fact is reflected in the much higher loss tangent values (Figure 3d) On the other hand, at low TCA concentration (**CTS_3_-TCA_5_**), *G*’ becomes slightly higher than *G*” in the whole frequency range studied, showing viscoelastic response. Moreover, a typical sol-gel transition response, with a crossover at medium frequencies, was exhibited by the **CTS_3_-TCA_0_** hydrogel. **CTS_3_-TCA_0_** and **CTS_3_-TCA_10_** present similar relative elasticity and **CTS_3_-TCA_5_** the lowest loss tangent values at low frequency (Figure 3d). 

**Figure 4:**
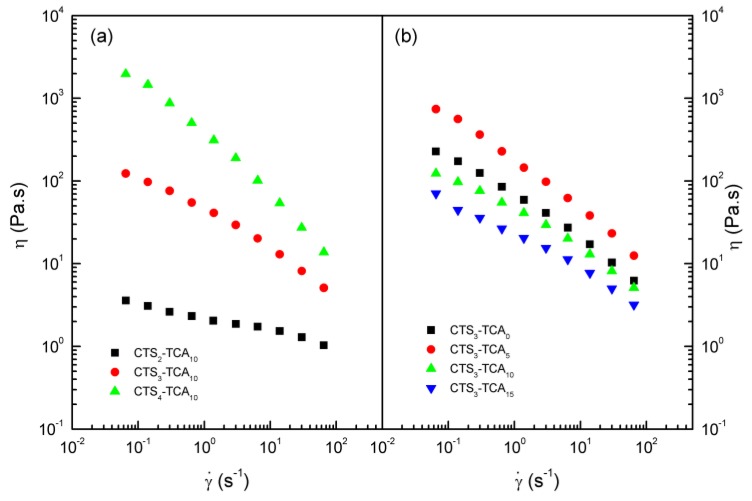
Viscous flow curves for **CTS_x_-TCA_y_** hydrogels studied, at 25 °C, as a function of (**a**) CTS concentration and (**b**) TCA concentration.

Viscosity values depend on CTS and TCA concentration in the same way as *G*′ and *G*″ do. Figure 4a,b depict the viscous flow behavior exhibited by selected hydrogels as a function of CTS and TCA concentration, respectively. The Williamson model fits fairly well this flow behavior in the shear rate range studied (R^2^ > 0.995) [23].

(1) $$\eta =\frac{\eta_{0}}{1+{\left(K\dot{\gamma }\right)}^{m}}$$

where η is the non-Newtonian viscosity; η_0_ is the zero-shear rate limiting viscosity; $\dot{\gamma }$ is the shear rate; m is a parameter related to the slope of the shear-thinning region; and *K* is a constant whose reciprocal coincides with the shear rate at which η = η_0_/2. 

The values of these fitting parameters are displayed in Table 1 for **CTS_x_-TCA_y_** hydrogels studied. Figure 4a depicts that viscosity clearly increases with CTS concentration, yielding higher values of η_0_, *K* and *m*. Moreover, regarding to TCA concentrations, **CTS_3_-TCA_5_** shows the highest values of viscosity and **CTS_3_-TCA_15_** the lowest ones.

The values of the fitting parameters to the Williamson model and loss tangent are shown in Table 2 for all **CTS_x_-TCA_y_** studied. As can be seen, viscosity values increase by increasing the CTS concentration and generally decrease by increasing the degree of crosslinking. Moreover, m values augment with CTS concentration indicating less pronounced shear-thinning characteristics of these hydrogels at low CTS concentrations but their flow behavior is almost unaffected by the degree of cross-linking. On the other hand, loss tangent decreases by increasing the CTS concentration and generally follows a similar trend with the degree of crosslinking.

#### 3.1.3. Modulated Temperature Differential Scanning Calorimetry (MTDSC) Studies of CTS-TCA Hydrogels

Modulated temperature differential scanning calorimetry (MTDSC) is the general term for DSC techniques where a non-linear heating or cooling rate is applied to the sample to separate the kinetic data from the thermodynamic ones. This technique finds numerous applications in the polymer field: for example, Higginbotham et al. [24] developed some thermosensitive polymer matrices based on *N*-isopropylacrylamide (NIPAAm). The thermosensitive hydrogels synthesized belong to the negative temperature-sensitive hydrogels category, with a lower critical solution temperature (LCST), and contract upon heating above the LCST. In general terms, the reversing heat-flow signal provided great sensitivity in the determination of LCST when compared with conventional DSC. In addition, we have studied polyHEMA hydrogels by thermal analysis. The transitions gel-sol were explored by MTDSC and the study was centered on the reversible heat-flow plot corresponding to transitions where the HEMA systems experienced a transformation from an arrangement with a higher heat capacity to one with a lower heat capacity [25]. As the phase transition temperature is a fully reversible transition [26,27], the reversible heat-flow curves reflect true gel-sol transitions and are, consequently, a better measurement of physical changes in these materials. Similarly we explored herein the phase transition temperature of the CTS-based hydrogels by MTDSC.

Some of the prepared CTS-based samples exhibited the typical transition gel-sol on thermo-reversible hydrogels and both, peak onset and peak maximum values were recorded in Table 3.

**Figure 5:**
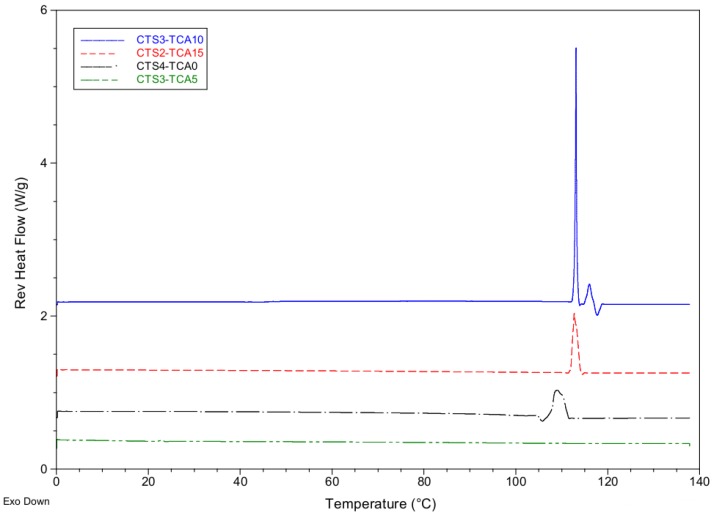
Reversible heat-flow plot and phase transition temperature from the temperature at the peak of the transition endotherm (°C) of **CTS_3_-TCA_10_** (solid line, in blue), **CTS_2_-TCA_15_** (short dash line, in red), and **CTS_4_-TCA_0_** (dash-dot line, in black) hydrogels established using modulated temperature DSC (MTDSC) overlaid with the reversible heat-flow versus temperature of **CTS_3_-TCA_5_** system (dash-dot line, in green).

Figure 5 displays the phase transition temperatures (°C) found at the peak of the transition endotherm and from the onset of the transition endotherm for several hydrogels. For samples **CTS_3_-TCA_10_** and **CTS_2_-TCA_15_** the transitions gel-sol took place in a very narrow interval confirming the thermo-reversible nature of **CTS_3_-TCA_10_** and **CTS_2_-TCA_15_** hydrogels. However, when the degree of crosslinking was reduced (for example, sample **CTS_3_-TCA_5_** compared to sample **CTS_3_-TCA_10_**), this transition was not observed. 

Additionally, an increase in CTS concentration helps the formation of gel-like systems since it decreases the amount of TCA needed as the CTS concentration increases. Nonetheless, if cross-linking is absent in the formulation (sample **CTS_4_-TCA_0_**), the transition endotherm shifts to lower values and does not show the typical sharp peak of truly cross-linked samples.

### 3.2. Diclofenac Loaded (CTS_x_-TCA_y_-DCNa_Z_) Formulations

Among the multitude of materials used to load and release drugs or biologically active species, positively charged polymers play an important role as nanocarriers with enhanced cellular uptake, improved encapsulation efficiency and high stability. Cationic polymershave been proven to be promising for the encapsulation and delivery of various anionic therapeutic compounds. Their charged nature allows complex formation with anionic molecules, such as diclofenac, DNA, and more [28]. Likewise, in drug delivery, the cationic polysaccharide CTS has been of special relevance as a carrier for various bioactive molecules due to its physico-chemical and biocompatible properties. Being hydro-soluble and positively charged, CTS interacts with negatively charged species in aqueous media and exhibits the singular feature of adhering to mucosal surfaces, a fact that makes it a useful biomaterial for mucosal drug delivery. CTS is able to promote transmucosal absorption of small polar drugs, including peptides, inducing a transient opening of the tight junctions of the cell membrane [29]. In addition, networks formed by non-covalent cross-linked CTS are largely used as DDS due to the possibility of drug diffusion [30].

Eight DDS named **CTS_x_-TCA_y_-DCNa_z_** were prepared with targeted final CTS concentrations 2%, 3% or 4% *w*/*w* and degree of crosslinking 0%, 5%, 10% or 15%. Diclofenac sodium (DCNa) was loaded into the hydrogel formulation so that the final *w*/*w* concentrations ranged from 0.5 to 2 %. In Table 2 and along the text “x” denotes CTS concentration (% *w*/*w*), “y” denotes the degree of cross-linking and “z” denotes DCNa concentration (% *w*/*w*). The influence of drug content, degree of cross-linking and CTS concentration in the drug release profiles and spreadability of the formulations were investigated.

#### 3.2.1. Scanning Electron Microscopy (SEM)

SEM is a valuable tool to evaluate the scaffold characteristics (pore size and morphology) of hydrogels and biological systems. In a previous study in which freeze dried method has been used with cells, it was demonstrated that this technique preserved the scaffold structure [31]. In this method the freezing step is critical since it fixes and determines the size of ice crystal in the scaffold structure. In the present work the hydrogels were freeze-dried following the procedure described by Nedel and co-workers [32] so that the water movement could be prevented by cryofixation. 

**Figure 6:**
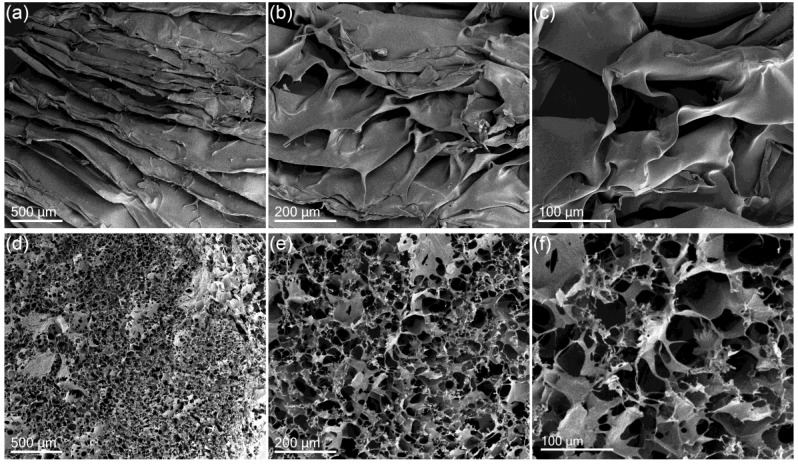
SEM images showing morphology of the as-synthesized unloaded chitosan-based hydrogel **CTS_3_-TCA_10_** (**a**–**c**) and loaded hydrogel **CTS_3_-TCA_10_-DCNa_1_** (**d**–**f**).

Figure 6 shows the SEM micrographs from the hydrogel **CTS_3_-TCA_10_** and DCNa-loaded hydrogel **CTS_3_-TCA_10_-DCNa_1_**. **CTS_3_-TCA_10_** did not show a porous scaffold but a well-defined lamellar structure probably due to the homogeneity of system (Figure 6a–c). Nonetheless, these results are opposite to those obtained by other authors; for example, when a bulky ionic cross-linker was used such as guanosine 5′-diphosphate (GDP), the SEM images showed a porous micro-structure [7] The packing of the bulky cross-linking agent in the hydrogel structure can cause some micro-irregularities responsible for the porous microstructure encountered. In addition, the method used for the preparation of the samples also exerts a great impact on the final microstructures. For example, and in contrast with the results presented herein, when the liquid-nitrogen fracture surfaces of some hydrogels prepared by ionic cross-linking were characterized using SEM after lyophilization, highly porous surfaces were encountered [5].

The spatial architecture of the prepared loaded CTS-hydrogels (Figure 6d–f), displayed highly porous scaffolds in contrast with non-loaded systems. Leroux and coworkers also found some porous structures in CTS-based DDS by SEM. They observed that drug loaded chitosan/glycerophosphate gels presented a highly porous structure after 24 h of exposure to a continuous flow of phosphate buffered saline in drug release experiments [19]. Unfortunately, no SEM image of drug-loaded formulations prior to release trials was taken to corroborate the origin of the highly porous scaffolds in their formulations.

In summary, the freeze-drying method used has proved to be a great method for fixation of CTS-based scaffolds for SEM studies since with the described lyophilization method, the pores, if present, were clearly distinguishable and the scaffold morphologies remained intact.

#### 3.2.2. Spreadability

In the design of polymeric pharmaceutical formulations for topical applications, several required product characteristics may be defined. Of those, spreadability of the product on the surface (skin or mucosal epithelium) and adhesion of the formulation when it is dealing with a mucosal surface are included. Such properties contribute to the final clinical efficacy of the product [33]. Mucoadhesion is controlled by the affinity of the DDS for the mucin glycoproteins of the mucus. Polysaccharides are very good mucoadhesive polymers because of their non-toxic nature, and capacity to bind to mucins through either electrostatic or hydrophobic interactions. The amine and hydroxyl groups of CTS are involved in its excellent mucoadhesive properties, leading, for example, to prolonged residence time in the gastrointestinal tract [3]. 

Evaluation of the spreadability of the formulations and voltaren emulgel^®^ was conducted for comparative purposes. Some formulations showed similar values of spreadability to the commercial hydrogel (see Table 2, **CTS_3_-TCA_15_-DCNa_1_** and **CTS_4_-TCA_10_-DCNa_1_**). It was observed that a reduction in CTS concentration led to low-structured formulations (**CTS_2_-TCA_10_-DCNa_1_**) and hydrogels, in accordance with their poor rheological properties. Conversely, with an increasing concentration of the anionic drug, a significant reduction in spreadability was observed. This could be explained by means of the pseudo-cross-linking effect of DCNa in CTS-based hydrogels.

#### 3.2.3. Diclofenac Sodium Release Studies

The exceptional biomedical properties of CTS have made it an ideal polymer for the preparation of muco-adhesive preparations that have been industrialized for ocular, nasal, buccal, gastrointestinal, and vaginal administrations with a great variety of drugs. Among them, DCNa, has been extensively used as a model anionic drug in numerous DDS formulations, particularly in CTS-based systems such as tablets, microspheres, microparticles and gels, due to its ionic linkage with CTS at acidic pH [29]. For example, the preparation of chitosan hydrogels bearing diclofenac, acetylsalycilic acid, and hydrocortisone acetate as anti-inflammatory drugs has been reported [34].

Additionally, macroporous CTS-nanographene oxide hybrid hydrogels have been shown to be effective adsorbent of this anti-inflammatory drug in wastewater [30]. However, as far as the authors are aware, an in-depth study of the influence of various parameters such as CTS concentration and drug content on the release profiles of an anionic drug in CTS-based formulations is still lacking in the literature.

**Figure 7:**
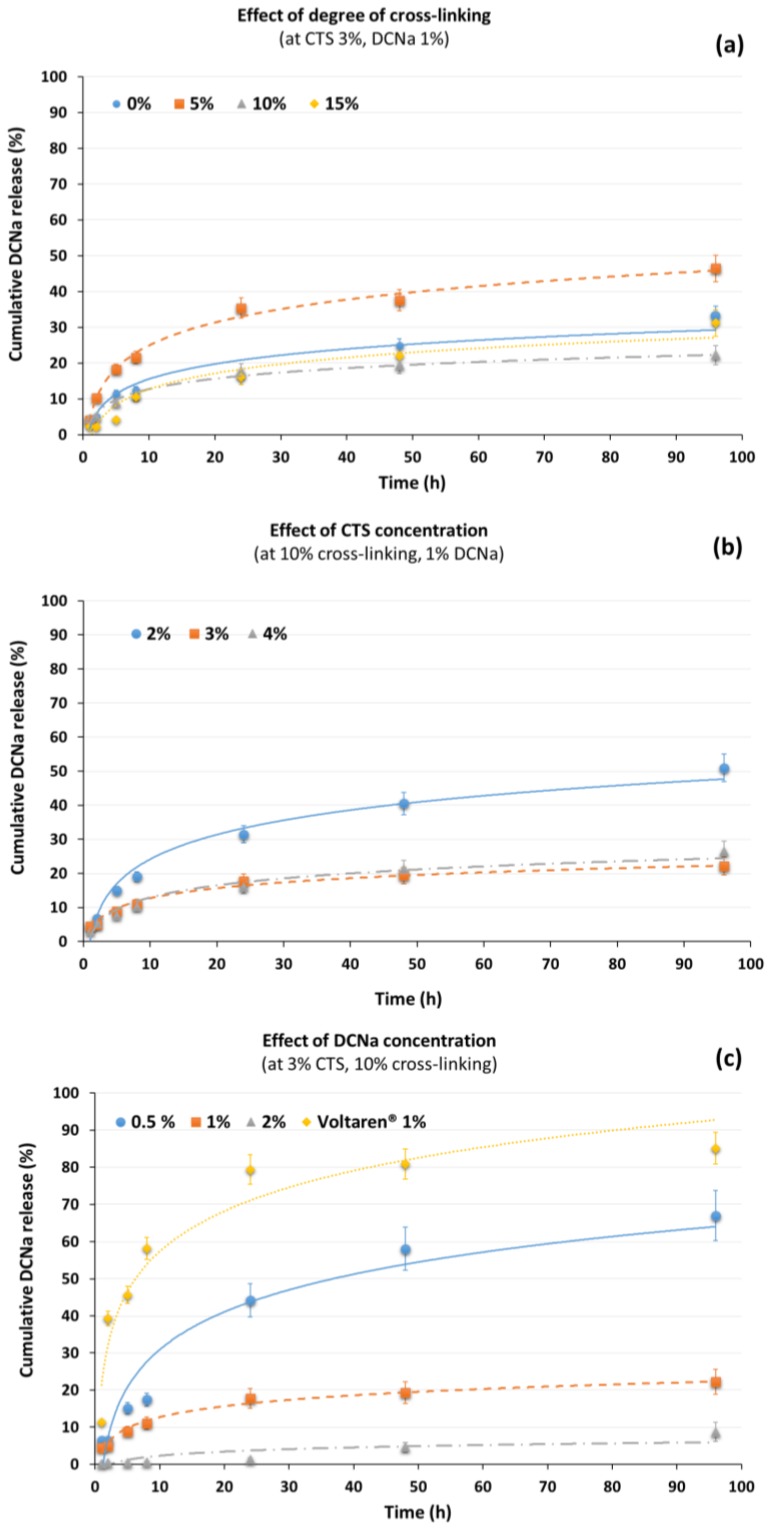
In vitro release profiles of diclofenac sodium (DCNa) from chitosan hydrogels in acetate buffer solution at pH 5.5 at 37 °C. Data were obtained from UV-Vis spectroscopy at 280 nm and reported as mean ± S.D from five independent experiments. (**a**) Effect of degree of cross-linking from non-cross-linked samples to 15% of cross-linked (CTS concentration 3%; DCNa concentration: 1%). (**b**) Effect of CTS concentration from 2% to 4% CTS concentration (degree of cross-linking: 10%; DCNa concentration: 1%). (**c**) Effect of DCNa concentration from 0.5% to 2% DCNa concentration (degree of cross-linking: 10%; CTS concentration: 3%).

The formulations prepared in the present work are recorded in Table 2. From the samples studied, it was observed that all the hydrogels were able to control the DCNa release for long periods of time at 37 °C in acetate buffer at pH 5.5 (Figure 7), close to the acidic pH which corresponds to the reduced pH microenvironment typical of cancerous cells (pH 6.5) [35]. The drug was released in a slow and sustained manner, ranging from 9% to 67% after 96 h. The influence of several factors, such as (a) the degree of crosslinking, (b) the CTS concentration, and (c) the drug content, on the release profiles has been studied.

It was first investigated the degree of cross-linking. The other two variables, concentration of CTS and drug content, were fixed at 3% and 1%, respectively. The results are recorded in Figure 7a. Regarding the degree of cross-linking, the percentage of DCNa delivered (after 96 h) ranged from 22% to 47% (samples **CTS_3_-TCA_10_-DCNa_1_** and **CTS_3_-TCA_5_-DCNa_1_**, respectively), displaying the formulation with a degree of crosslinking of 10% the most controlled release pattern. Surprisingly, a degree of crosslinking greater than or less than 10% unequally affected the release of DCNa. Thus, for example, when the crosslinking was 5%, a boost in the drug release was observed, being the latter even greater than those results obtained from CTS-based dispersions without TCA in the formulation. It is hypothesized that the presence of TCA alters, to a certain extent, the hydrogen bonds that keep the CTS chains tight together. Thus, more free volume can be found, which allows a quicker diffusion of the drug to the medium. 

When the concentration of CTS was investigated (Figure 7b), the degree of cross-linking was set at 10% for all samples and the concentration of the drug in the formulation was fixed at 1%. It was observed that the hydrogels displayed improved control over the drug release when the CTS concentration was 3% or higher (with percentages of DCNa release below 25% after 96 h). In contrast, CTS concentrations inferior to 3% conducted to a lower drug-controlled efficiency, causing a two-fold increase in drug release. 

Lastly, the amount of drug in the formulation exerted a significant impact on the kinetics of drug release (Figure 7c). For the samples studied, the CTS concentration and degree of cross-linking were set at 3% and 10%, respectively and the drug concentration ranged from 0.5% to 2%. For comparative purpose, Voltaren emulgel^®^ from Novartis Pharmaceuticals was investigated, with a 1% concentration of diclofenac diethylammonium in its formulation. In all cases, except for the commercial product with a boosted release from the first few hours, the higher the diclofenac content in the prepared samples, the lower the DCNa released from the formulations, generating well-controlled delivery formulations. With a p*K*a of 4.1, the non-steroidal anti-inflammatory agent diclofenac is anticipated that would mostly be in its anionic form under the experimental conditions, interacting ionically with the ammonia residues of CTS. It should be noted that the ionized carboxylate group equally distributes its negative charge between the two oxygen atoms from the functional group and, therefore, ionic attractions with two ammonia groups could be possible, acting the carboxylate group as a pseudo-cross-linker in the dispersion. These hypotheses can also explain the reduction in spreadability of samples with the same CTS content and degree of crosslinking (see Table 2, samples **CTS_3_-TCA_10_-DCNa_0.5_**, **CTS_3_-TCA_10_-DCNa_1_** and **CTS_3_-TCA_10_-DCNa_2_**). Consequently, at the highest DCNa concentration studied, the release of the drug is almost totally impeded, with values lower than 10% in 96 h.

## 4. Conclusions

This study reports the preparation and use as drug carriers of a new family of eco-friendly reversible chitosan hydrogels highly compatible with biological compounds. The hydrogels have been successfully prepared by ionic cross-linking with propane-1,2,3-tricarboxylic acid (tricarballylic acid, TCA). 

The rheological properties of CTS_x_-TCA_y_ hydrogels could be modulated by modifying the CTS and TCA concentrations; thus, low CTS concentrations produced liquid-like viscoelastic dispersions in contrast to the strong gel-like features found at high CTS, whereas an intermediate response was observed at 3% *w*/*w* CTS. On the contrary, at high TCA concentration the dispersions mainly exhibited a viscous response, whereas at an intermediate TCA concentration, *G*’ coincided with *G*” or typical sol-gel transition responses were found. Viscosity values increased with increasing the CTS concentration and generally decreased with increasing the degree of crosslinking. 

The thermal transitions gel-sol were investigated by MTDSC and the study was focused on the reversible heat-flow plot. Some transitions gel-sol took place in a very narrow interval confirming the thermo-reversible nature of **CTS_3_-TCA_10_** and **CTS_2_-TCA_15_** hydrogels. The presence of a broader peak and the shift of the transition endotherm to lower values was observed when the cross-linker was lacking in the formulation (sample **CTS_4_-TCA_0_**). The spatial architecture of non-loaded and loaded CTS-hydrogels were studied and they markedly differed in their microstructure, displaying the latter highly-porous scaffolds in contrast with non-loaded systems. 

Eight diclofenac sodium formulations were prepared and the relationships between polymer concentration, drug loading and degree of cross-linking with the release profiles were evaluated. They displayed a well-controlled drug release strongly dependent on the formulation composition and cumulative drug release under physiological conditions for 96 h varied from 8% to 67%. For comparative purpose, a commercial formulation, Voltaren emulgel^®^ from Novartis Pharmaceuticals, with 1% of diclofenac concentration, was also investigated. In conclusion, the preparation method of CTS-based dispersions presented herein provides a simple and easy method to tailor-made controlled-release systems with improved rheological properties. 

## Figures and Tables

**Table 1:** Concentration and degree of cross-linking of the 12 CTS-based hydrogels prepared. Rheological parameters.

Hydrogel	CTS		TCA		pH	Rheological Properties ^1^			
CTS_x_-TCA_y_	mg CTS	Conc. (% *w*/*w*)	mg TCA	Degree Xr (%)		η_o_ (Pa.s)	K (s)	m	Tan δ (1 rad/s)
**CTS_2_-TCA_0_**	200	**2%**	-	**0**	5.2	5	0.13	0.6	2.57
**CTS_3_-TCA_0_**	300	**3%**	-	**0**	5.3	496	20.18	0.6	1.19
**CTS_4_-TCA_0_**	400	**4%**	-	**0**	5.3	5616	17.44	0.85	0.62
**CTS_2_-TCA_5_**	200	**2%**	2.6	**5**	5.3	9	0.095	0.55	3.08
**CTS_3_-TCA_5_**	300	**3%**	3.8	**5**	5.2	1123	8.57	0.7	0.81
**CTS_4_-TCA_5_**	400	**4%**	5.1	**5**	5.2	2061	7.77	0.82	0.59
**CTS_2_-TCA_10_**	200	**2%**	5.1	**10**	5.3	5	0.47	0.37	5.14
**CTS_3_-TCA_10_**	300	**3%**	7.7	**10**	5.3	155	4.57	0.6	1.47
**CTS_4_-TCA_10_**	400	**4%**	10.3	**10**	5.2	3745	13.24	0.83	0.68
**CTS_2_-TCA_15_**	200	**2%**	7.7	**15**	5.3	3	0.08	0.59	2.88
**CTS_3_-TCA_15_**	300	**3%**	11.5	**15**	5.2	100	10.97	0.49	1.77
**CTS_4_-TCA_15_**	400	**4%**	15.4	**15**	5.2	2151	8.41	0.82	0.86

^1^ Williamson’s model parameters and loss tangent at 1 rad/s for CTS_x_-TCA_y_ hydrogels studied.

**Table 2:** Composition and evaluation of drug formulations.

Formulation Code	CTS Conc (% *w*/*w*)	Degree *X*r (%)	Drug Content (%)	Spreadability (Diameter, cm)			Drug Release (%)		
				*t* = 1 min	*t* = 30 min	Diameter (%)	*t* = 5 h	*t* = 24 h	*t* = 96 h
**CTS_3_-TCA_0-_DCNa_1_**	3	--	1	4.4	6	36	11	17	33
**CTS_3_-TCA_5-_DCNa_1_**	3	5	1	4.2	7.3	74	18	35	46
**CTS_3_-TCA_10-_DCNa_1_**	3	10	1	4.4	5.2	18	9	18	22
**CTS_3_-TCA_15-_DCNa_1_**	3	15	1	4.1	5.70	39	4	16	31
**CTS_2_-TCA_10-_DCNa_1_**	2	10	1	5.4	10.6	96	15	31	51
**CTS_3_-TCA_10-_DCNa_1_**	3	10	1	4.4	5.2	18	9	18	22
**CTS_4_-TCA_10-_DCNa_1_**	4	10	1	3.6	4.80	33	8	16	26
**CTS_3_-TCA_10-_DCNa_0.5_**	3	10	0.5	3.9	6.4	64	15	44	67
**CTS_3_-TCA_10-_DCNa_1_**	3	10	1	4.4	5.2	18	9	18	22
**CTS_3_-TCA_10-_DCNa_2_**	3	10	2	2.3	2.5	9	0	1	9
**Voltaren emulgel**	--	--	1	4.30	5.70	33	46	79	85

**Table 3:** Thermal properties of selected CTS-based hydrogels from modulated temperature differential scanning calorimetry (MTDSC).

Sample	MTDSC		
	Peak Temp. ^a^	Onset Temp. ^b^	Rev. Heat Flow ^c^
CTS_2_-TCA_15_	112.71	112.09	28.68
CTS_3_-TCA_10_	113.10	112.85	96.19
CTS_3_-TCA_5_	--	--	--
CTS_4_-TCA_0_	109.01	107.71	25.39

^a^ Peak Temp.: Phase transition temperature from the temperature at the peak of the transition endotherm (°C); ^b^ Onset Temp.: Phase transition temperature from the onset of the transition endotherm (°C); ^c^ Rev. Heat Flow: Reversible heat flow (J/g).
